# The role of hemoadsorption in septic shock: toward a personalized approach

**DOI:** 10.1186/s13054-026-06135-1

**Published:** 2026-06-13

**Authors:** Zsolt Molnar, Tamas Toth, Claudio Ronco, Jean-Louis Teboul, Vedran Premužić, Steffen Mitzner, Daniel De Backer, Gerd Klinkmann, Konstanty Szuldrzynski, Fabio Silvio Taccone, Gabriella Bottari, Manu L. N. G. Malbrain, V. Marco Ranieri

**Affiliations:** 1 Center for Translational Medicine, Semmelweis University, Budapest, Hungary; 2 Department of Anesthesiology and Intensive Therapy, Semmelweis University, Budapest, Hungary; 3 Department of Anesthesiology and Intensive Therapy, Poznan University of Medical Sciences, Poznan, Poland; 4 Department of Anesthesiology and Intensive Therapy, Bajcsy-Zsilinszky Hospital, Budapest, Hungary; 5 International Renal Research Institute of Vicenza (IRRIV), Vicenza, Italy; 6 Department of Medicine (DIMED), Università degli Studi di Padova, Padova, Italy; 7 Paris-Saclay Medical School, Paris-Saclay University, Le Kremlin-Bicêtre, France; 8 Department of Nephrology, Hypertension, Dialysis and Transplantation, University Hospital Center Zagreb, Zagreb, Croatia; 9 School of Medicine, Catholic University of Zagreb, Zagreb, Croatia; 10 Division of Nephrology, Center for Internal Medicine, Rostock University Medical Center, Rostock, Germany; 11 Department of Intensive Care, CHIREC Hospitals, Université Libre de Bruxelles, Brussels, Belgium; 12 Department of Anaesthesiology and Intensive Care Medicine, University of Rostock, Rostock, Germany; 13 Department of Extracorporeal Immunomodulation, Fraunhofer Institute for Cell Therapy and Immunology, Rostock, Germany; 14 Department of Anaesthesiology and Intensive Care, National Medical Institute of the Ministry of the Interior and Administration, Warsaw, Poland; 15 Department of Intensive Care, Hôpital Universitaire de Bruxelles (HUB), Université Libre de Bruxelles (ULB), Brussels, Belgium; 16 Pediatric Intensive Care Unit Children Hospital Bambino Gesù, Rome, Italy; 17 First Department of Anaesthesiology and Intensive Therapy, Medical University of Lublin, Lublin, Poland; 18 International Fluid Academy, Lovenjoel, Belgium; 19 Medical Data Management, Medaman, Geel, Belgium; 20 Department of Precision-Regenerative Medicine and Jonic Area, Section of Anesthesiology, University of Bari “Aldo Moro”, Bari, Italy; 21 Department of Anesthesia and Critical Care, Policlinico di Bari, Bari, Italy

**Keywords:** Hemoadsorption, Septic shock, Sepsis, Personalised medicine, Extracorporeal blood purification, Hyperinflammation, Cytokine storm, Endothelial protection

## Abstract

A dysregulated host response to infection is central to the pathophysiology of sepsis and may culminate in life-threatening organ dysfunction. Given that this process is largely characterized by concurrent pro- and anti-inflammatory activation, immunomodulatory strategies have long been explored in sepsis research. Among these, extracorporeal removal of circulating cytokines, inflammatory mediators and other soluble factors through non-specific hemoadsorption with macroporous styrene-divinylbenzene sorbents has been proposed as a potential therapeutic approach. Its adoption into clinical practice has largely been based on pathophysiological considerations rather than on evidence from large, well-designed randomized clinical trials. Over the past 15 years, most of the available evidence has been predominantly derived from small, single-center cohorts, reports from registries and heterogeneous prospective studies with substantial variability in patients’ selection, timing, and treatment intensity. In addition, the precise mechanisms of action of hemoadsorption remain incompletely understood. Although several meta-analyses have attempted to synthesize the existing data, the overall quality and heterogeneity of the included studies limit the strength and reliability of their conclusions. As a result, current guideline recommendations are largely based on expert opinions rather than high-certainty evidence. This position statement aimed to provide a concise overview of the biological rationale, current evidence, and contemporary clinical practice related to hemoadsorption in critically ill patients.

## Background

Sepsis is defined as a life-threatening organ dysfunction caused by a dysregulated host response to infection [1]. The most severe manifestation of sepsis is septic shock, which results in cardiovascular failure. A key mechanism of septic shock is the imbalance between pro- and anti-inflammatory pathways. During the hyperinflammatory phase, the uncontrolled and excessive release of cytokines, complement factors, and other mediators can generate a self-perpetuating vicious cycle, which may lead to endothelial injury and glycocalyx shedding, resulting in increased vascular permeability, tissue oedema and microcirculatory dysfunction [2–4]. These phenomena contribute to progressive fluid accumulation and progression of organ dysfunction [5]. Concurrently, vasoplegia with impaired vascular responsiveness to vasopressors exacerbates circulatory failure and result in tissue hypoxia, complicating the clinical management of septic patients [6]. This condition has been associated with mortality rates up to 70% despite guideline-based standard therapy [7]. Given the central role of immune dysregulation in the pathophysiology of septic shock, together with the limited effectiveness of conventional supportive therapies in reversing this process, adjunctive strategies aimed at modulating the host response have attracted increasing interest. In this context, extracorporeal hemoadsorption using macroporous styrene–divinylbenzene sorbent cartridges may contribute to restoring immune homeostasis and improving hemodynamic stability in selected patients with septic shock [8, 9].

## Complexity and heterogeneity of sepsis phenotypes

Sepsis is not a uniform disease but a syndrome encompassing multiple clinical and biological phenotypes. Advances in computational approaches and transcriptomic profiling have identified sepsis phenotypes that may explain variability in treatment responses and differences in patient outcomes [10]. Seymour et al. [10] categorized sepsis into four distinct phenotypes; the α phenotype, characterized by a younger age, with a lower prevalence of comorbidities and a reduced mortality rate; the β phenotype, characterized by older individuals afflicted with chronic diseases and renal dysfunction; the γ phenotype, characterized by pronounced systemic inflammation and respiratory failure; the δ phenotype, associated with hepatic dysfunction and the highest mortality risk. However, applying these categories is often complicated because of the existence of significant phenotypic overlap within the same patient [11]. Other phenotypes could easily be identified or added like those with profound, global increased permeability syndrome, or those with vasoplegia, or those with myocardial depression or right heart failure [12–14]. Furthermore, the immunological landscape of sepsis is highly dynamic, resulting in patients often shifting between phenotypes [15, 16].

Identifying the hyperinflammatory phenotype at the bedside has historically been challenging. The recent PHIND study by Reddy et al. [17] demonstrated that real-time identification of inflammatory subphenotypes is feasible using a bedside immunoanalyser that quantifies plasma IL-6 and soluble TNFR1. In this prospective multicenter cohort study, 18% of patients presented a hyperinflammatory subphenotype, which was independently associated with a significantly higher 60-day mortality (51% vs. 28%). However, evidence suggests these subphenotypes can be dynamic. The proportion of hyperinflammatory patients may decrease over the first 72 h due to natural disease evolution or treatment effects. These findings underscore the importance of rapid biomarker evaluation to successfully make decisions [17].

This heterogeneity challenges the conventional “one-size-fits-all” approach reflected in current guidelines, as interventions advantageous for hyperinflammatory patients profiles could be ineffective or even harmful in patients with immunosuppressed profiles [18, 19]. Similarly, two phenotypes with opposite responses to steroids were described in patients with Acute Respiratory Distress Syndrome (ARDS) [20]. Additionally, the interventions themselves- such as administration of corticosteroids- can actively alter patients’ phenotypes [21, 22]. This biological diversity also highlights the importance of individualized, phenotype-driven therapeutic strategies [23–25]. Recently, the ImmunoSep randomized clinical trial supported this concept by demonstrating that precision immunotherapy tailored to specific immune dysregulations, such as macrophage activation–like syndrome or sepsis-induced immunoparalysis, improved organ dysfunction compared to placebo [26].

### Hemoadsorption in septic shock

According to the consensus reached at the 30th Acute Disease Quality Initiative (ADQI) [27] and the 2024 Rome meeting [28], HA is the passage of blood through a sorbent containing cartridge or a hemofilter for selective or broad-spectrum solute removal via direct contact of the blood with the sorbent material or the filter membrane surface. This process is primarily mediated by physical adsorption, utilizing van der Waals forces (i.e. those arising from electrostatic interactions between partial charges that develop when the electron distribution around atoms or molecules becomes uneven), hydrophobic interactions, and ionic binding to capture target molecules [27]. The principles of HA (mass transfer zone, isotherms and flow dynamics) and cartridge-specific features (surface area, the adsorption capacity and specific target molecules removal) explain the different mechanisms (i.e. cytokine removal, endothelial protection, thrombo-inflammation modulation) that may influence clinically relevant outcomes, such as vasopressor reduction, shock reversal and reduced mortality [29].

HA devices remove circulating pathogen-associated molecular patterns (PAMPS), damage-associated molecular patterns (DAMPS), and inflammatory mediators through adsorption [8]. The conceptual aim is not a complete suppression of the immune response, but rather rebalancing homeostasis by dampening the “toxic peak” of hyperinflammation [8, 30], highlighting the importance of the concentration-driven mechanism of these devices [31]. From the theoretical standpoint, potential therapeutic benefits may result from control of excessive hyperinflammation [32], modulating thrombo-inflammation [33], and protection of the endothelium and stabilization of the glycocalyx [34, 35]. This phenomenon has been described as the Global Increased Permeability Syndrome (GIPS) and contributes to the development of the Fluid Accumulation Syndrome (FAS), characterized by progressive interstitial edema, tissue congestion, venous hypertension, increased compartmental pressures (intrathoracic, intra-abdominal) and impaired organ function. In critically ill patients, positive fluid balance and extravascular fluid accumulation are consistently associated with worse outcomes, including prolonged mechanical ventilation, renal dysfunction, and increased mortality [36].

Endothelial protection may lead to reduction of vasoplegia, improved microvascular perfusion, improved vascular responsiveness [37], reversal of shock [38], facilitation of subsequent negative fluid balance by limiting capillary leak [39], and eventually restoration and improvement of organ function [40]. Experimental data demonstrate that these devices can reduce circulating pro- and anti-inflammatory cytokines [32], while clinical observational studies report decreased vasopressor requirements and trends toward hemodynamic stabilization [41]. Although survival benefits remain controversially discussed, these biological and clinical signals provide the rationale for ongoing investigation into HA as an adjunctive therapy in refractory septic shock.

The rationale for HA is strongest in patients with evidence of hyperinflammation, such as refractory septic shock with persistent vasoplegia despite adequate source control and resuscitation [42], or evidence of sustained high cytokine release indicated by high levels of elevated inflammatory markers such as IL-6, CRP, or procalcitonin [43, 44]. Recent registry analyses and a meta-analysis focused on septic shock aggregating 9 studies and including 744 patients suggest that specific subgroups, particularly those with high vasopressor dependency and excessive inflammation, may experience vasopressor reduction and improved hemodynamic stability during HA [45], with signals indicating also a potential survival benefit. Although IL-6 has been proposed as a useful biomarker to guide patient selection for hemoadsorption, its clinical application remains limited by variable kinetics, lack of standardized thresholds, and restricted assay availability, suggesting that an approach based on the measurement of multiple biomarkers (i.e. ferritin, HLA-DR expression), and/or procalcitonin kinetics, may provide a more robust framework for identifying appropriate candidates [13, 46]. Consequently, accurate identification of potential responders using clinical features and biomarker profiles is essential to guide therapy and optimize outcomes.

Despite these pathophysiological considerations and encouraging physiological signals, current evidence syntheses have not demonstrated a consistent benefit of HA on patient-centered outcomes. Contemporary international guidelines for the management of sepsis and septic shock consider the evidence around unspecific HA therapies as not sufficient for the provision of a recommendation, neither positive nor negative [23]. Similarly, recent systematic reviews, meta-analyses and propensity matched analyses largely based on small, heterogeneous, and methodologically limited studies, have failed to show a survival advantage and have highlighted substantial uncertainty regarding efficacy. As a result, HA is currently regarded as an adjunct under ongoing investigation, and its use should be personalised to selected patients within structured protocols or research settings until higher-quality randomized evidence becomes available [23, 47–50]. Although hemoadsorption is generally considered safe, potential limitations and risks—including unintended removal of therapeutic drugs such as antibiotics, adsorption of beneficial molecules such as albumin, anticoagulation-related complications, circuit clotting, and questions regarding cost-effectiveness—should be acknowledged when evaluating its clinical use [51].

The clinical trajectory of HA has largely followed a “learning-by-doing” paradigm. The intervention entered routine practice in the absence of adequately powered randomized controlled trials, relying instead on experimental evidence [52] and pathophysiological plausibility [53]. Despite this limited evidentiary foundation, its use in septic shock disseminated rapidly worldwide, and the resulting bedside experience has been described in multiple reports [45]. Moreover, the precise mechanism(s) of action remain incompletely elucidated. Although recent meta-analyses have attempted to consolidate these findings, their inferences are constrained by pronounced clinical heterogeneity, variability in underlying disease states, and the generally low quality of the included data, rendering the resulting conclusions in part unconvincing and, in several instances, potentially even misleading. Consequently, guideline statements have often been driven more by expert consensus than by robust, definitive evidence.

A major challenge in the interpretation of clinical trial results and the design of treatment strategies is the discrepancy between the need for individualized treatment decisions and the “one size fits all” approach reflected in many national guidelines [23, 24]. In Germany, for example, recent guideline recommendations for blood purification have been generalized across heterogeneous critically ill populations, without sufficient distinction between different patient phenotypes [24]. This becomes particularly problematic when sepsis and septic shock are considered jointly, despite their distinct pathophysiological profiles and therapeutic needs. As illustrated by the divergent guideline recommendations for hydrocortisone and antibiotics in sepsis versus septic shock, uniform guidance may fail to capture relevant differences in disease severity and response to therapy, limiting clinical applicability and potentially obscuring patient benefit [23, 24]. Negative guideline recommendations, if interpreted too rigidly, risk discouraging the use of potentially beneficial interventions in selected critically ill patients and may inadvertently create therapeutic gaps in cases of refractory shock.

## The heterogeneity of hemoadsorption techniques

Hemoadsorption is not a singular intervention; rather, it encompasses a diverse spectrum of devices with distinct adsorptive properties [54, 55]. While most of the literature focuses on Cytosorb which targets unspecific cytokine removal, several other technologies are available [56]. Jafron (e.g., HA330 and HA380), similar to Cytosorb, targets a broad spectrum of inflammatory mediators and have demonstrated signals of efficacy in reducing hospital mortality and vasopressor requirements in recent network meta-analyses [56, 57]. Based on pathophysiological rationale, these techniques are likely beneficial for patients suffering uncontrolled hyperinflammatory states [54]. Conversely, Polymyxin B hemoperfusion is specifically designed to selectively bind circulating endotoxin; thus, this approach could be beneficial for patients with Gram-negative bacterial infections and septic shock [55]. The recently published Tigris trial supports this concept in highly selected patient populations [58]. Additionally, the oXiris membrane aims to combine continuous renal replacement therapy with both endotoxin and broad cytokine adsorption capabilities. Consequently, these properties may be beneficial for patients with concomitant renal failure and a hyperinflammatory state [55, 56].

However, available data reveal significant knowledge gaps and potential risks associated with these treatments, while conflicting results highlight the dangers of a one-size-fits-all approach [54]. Broad-spectrum adsorption carries the risk of the unintended clearance of therapeutic drugs [54]. Furthermore, the adsorptive capacity of these cartridges is limited, potentially leading to the dangerous phenomenon of “desorption,” wherein previously adsorbed harmful molecules are - albeit quantitatively to a lesser extent - released back into the circulation [54]. These mechanisms may contribute to the negative outcomes or the premature termination of trials, such as COMPACT-2 [59]and ROMPA [60], due to increased mortality or futility [54]. Such negative results do not invalidate the usefulness of hemoadsorption therapies; rather, they powerfully demonstrate that applying uniform extracorporeal interventions across biologically heterogeneous populations, such as patients with septic shock, is predisposed to fail [54]. Moreover, it is important to note that the technique under investigation in both these trials was the Coupled Plasma Filtration Adsorption device (CPFA; Bellco, Italy), which – in contrast to modern whole blood hemoadsorbers – due to limited hemocompatibility involved plasma separation prior to the adsorption process.

Hemoadsorption strategies must shift from treatments based merely on mechanistic plausibility and physiological rationale toward precise, prognostic, and predictive enrichment-guided therapies [54, 55]. Under this paradigm, patients with confirmed Gram-negative endotoxemia might benefit most from targeted Polymyxin B hemoperfusion, whereas a patient whose shock is driven predominantly by a massive cytokine storm might be a better candidate for broad-spectrum adsorbers like CytoSorb or Jafron [54, 55, 58]. Ultimately, the most rational therapeutic aim is to integrate immune monitoring techniques to match the specific adsorber to the underlying “treatable trait,” maximizing efficacy and safety [54, 61].

## Position on clinical practice at the bedside

**Fig. 1:**
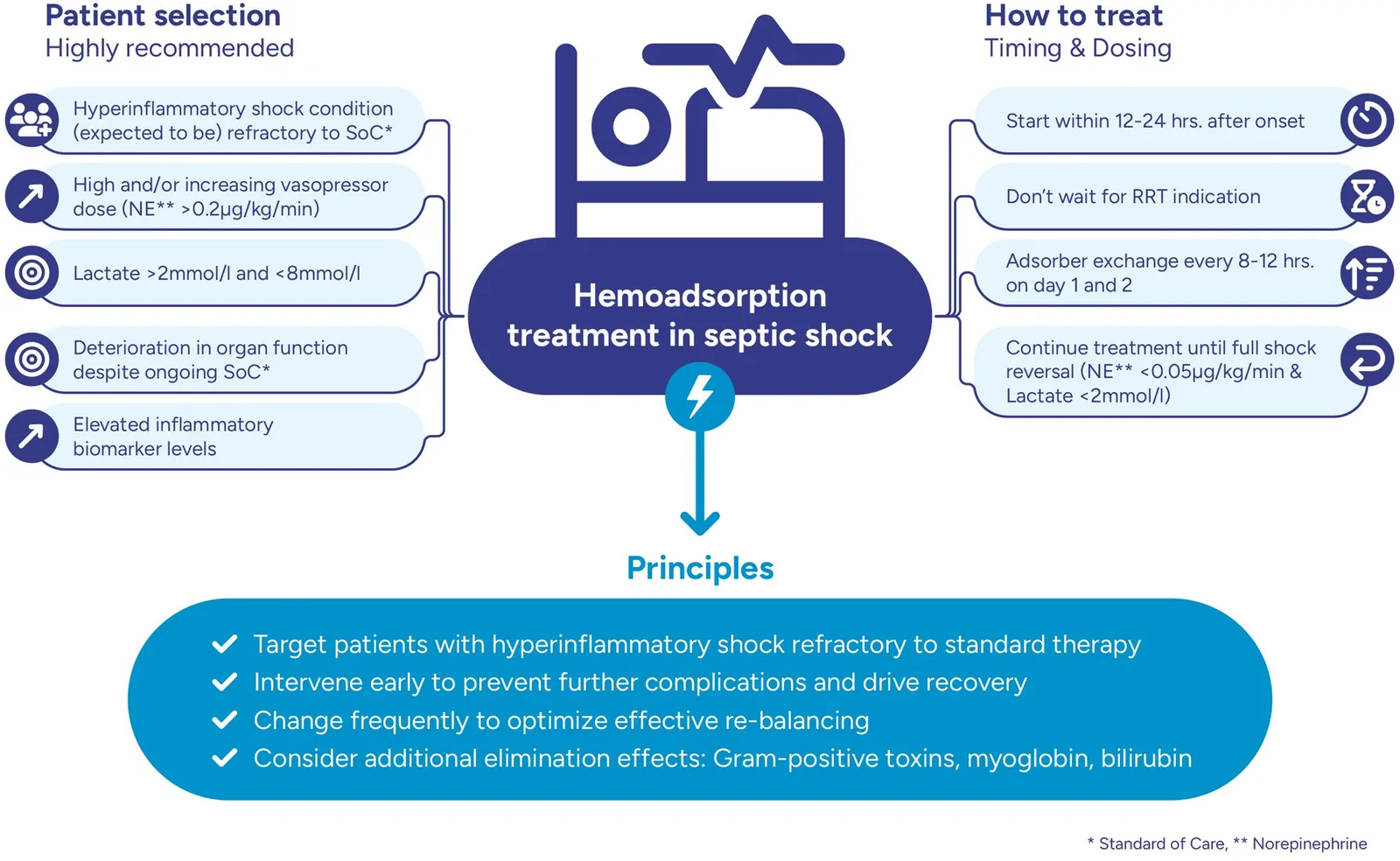
Proposed optimal clinical settings if HA is to be implemented

Based on the accumulated clinical experience and pathophysiological understanding, HA could be primarily considered an adjunctive treatment in patients with a hyperinflammatory phenotype of septic shock, who do not respond to standard medical therapy [56]. Three main issues require careful consideration for successful treatment: patient selection, timing and dosing, summarized in Fig. 1.

While high-quality evidence from large randomized controlled trials is currently lacking, available data suggest that if HA therapy is to be implemented, early initiation may be considered, ideally within the first 12 h and no later than 24 h after the diagnosis of septic or vasoplegic shock. The earlier phase might be the most appropriate clinical setting to maximize effectiveness and improve patient outcomes [8]. However, in real-world settings, early initiation of HA treatment is frequently hindered by delays in recognizing sepsis and diagnostic difficulties [62]. Considering these diagnostic and logistical limitations, there is no clear cut-off for optimal timing, nevertheless the likelihood of success decreases over time [63]. This decrease probably occurs concomitantly with the establishment of structural, irreversible organ failure, which replaces the purely hyperinflammatory and potentially still reversible state seen in the early phase of the syndrome [64]. In summary, if HA therapy is utilized, current clinical experience suggests that it should be initiated as early as possible to maximize potential effectiveness.

Regarding dosing and duration, adsorber replacement every 8–12 h is commonly suggested until hemodynamic improvement is achieved, for example, when norepinephrine (base) requirements fall below 0.05 µg/kg/min [8]. However, treatment duration should be strictly individualized based on the patient’s clinical evolution. When endogenous production is predominant, maintaining treatment duration is critical to prevent rebound phenomena [65].

Several randomized controlled trials and observational studies have reported a neutral or even negative impact of HA use in critically ill patients [66]. Negative study results are often associated with the inclusion of patients with less severe or no hyperinflammation, who by definition should not be expected to benefit from the treatment [67, 68].

The accumulating evidence suggests that unfavorable outcomes often reflect methodological shortcomings such as imprecise patient selection, delayed initiation, and inconsistent dosing. However, therapeutic effects may be context-specific or even absent in broad, unselected sepsis populations [45]. This approach highlights the importance of prognostic and predictive enrichment factors to identify those patients who benefit the most from hemoadsorption treatment. The recently published results of the TIGRIS trial [58] validated this concept. Targeting specific patients characterized by hemodynamic instability, multi-organ failure and increased but not excessive endotoxin activity showed high posterior probability of reduced mortality. These results show that combining rigorous trial design with precise prognostic and predictive patient enrichment can uncover beneficial therapeutic effects that would otherwise be undetected.

Evidence suggests that heterogeneity in clinical indications, initiation criteria, and integration into multimodal treatment strategies may obscure potential benefits of HA therapy [69]. Therefore, careful identification of suitable patient subgroups and standardized utilization protocols are crucial to accurately assess the therapeutic value of these techniques [50, 67, 70, 71]. Furthermore, traditional randomized controlled trials often struggle to capture benefits in specific subgroups due to sepsis heterogeneity, complex pathophysiology, and numerous co-interventions.

Furthermore, interpreting the impact of HA on all-cause mortality in clinical trials is confounded by different factors, often related not only to the acute sepsis episode but also to underlying comorbidities or frailty [72, 73]. This connects to the ‘treatable traits’ concept proposed by Bos et al. [61], which emphasizes that during the treatment it is important to focus on observable, modifiable biological abnormalities to improve treatment success. This strategy, which effectively utilizes predictive enrichment, was also recently investigated and shown to be beneficial in the ImmunoSep trial [26]. However, researchers must recognize that even an effective intervention targeting specific traits such as hyperinflammatory responses or severe vasoplegia may have a limited effect on all-cause mortality. Therefore, to better define the exact clinical role of HA, ongoing and future research must evolve. Future efforts should prioritize detailed physiological studies and biomarker-driven enrichment strategies to identify the most promising therapeutic tools [64]. Additionally, constructing large-scale biobanks and comprehensive registry-based studies, alongside employing innovative methodologies such as adaptive platform trials, will be crucial for generating robust evidence in this patient population [74].

Indicators of futility include progressive multi-organ failure despite maximal supportive care, persistent immunoparalysis, or no need for vasopressors, inotropic therapy, or mechanical circulatory support. Continuing HA in these patients should be avoided to prevent unnecessary resource use and potential harm [69].

The phenotypes that could potentially benefit the most are those with high or increasing vasopressor requirement (norepinephrine > 0.2 µg/kg/min or second vasopressor), moderate hyperlactatemia (serum lactate > 2 mmol/L and < 8 mmol/L) and platelet count > 100 × 10^9^/L [37, 42], accompanied by elevated inflammatory marker levels. Defining the right patient population in real-time remains challenging despite all efforts, but the recently published Delphi consensus about Clinical criteria for the definition of refractory septic shock by the Society of Critical Care Medicine (SCCM) and the European Society of Intensive Care Medicine (ESICM) provides a standardized framework to help identifying this phenotype [75], although the threshold vasopressor level of 0.5 µg/kg/min mentioned in the definition might be too high and could jeopardize the concept of “as early as possible start of HA”. Therefore, accurate identification of potential responders is essential, relying not only on clinical features like high vasopressor dependency but ideally also on the change in biomarker levels. However, identifying the latter needs further studies including pre- and post-cartridge measurements, to identify appropriate candidates and detect cartridge saturation [76]. HA therapy can be used as a standalone treatment [41] or as a complementary adjunct to Continuous Renal Replacement Therapy (CRRT). It should be noted that waiting for the development of acute kidney injury requiring CRRT [27, 77] to justify combination therapy may result in undue delay of HA initiation and loss of its potential benefit, keeping in mind the clinical risks of early catheterization and anticoagulation, as well as the financial burden of the treatment.

Complementary to this procedural optimization, integrating upstream and downstream monitoring of cytokine levels can refine therapy control and enable tailoring to individual patient needs [78]. If HA is utilized, treatment may be continued until shock reversal, as defined by norepinephrine (base) < 0.05 µg/kg/min and serum lactate < 2 mmol/L is achieved [8]. Additionally, patients suffering from severe Gram-positive infections may benefit from concomitant direct staphylococcus and streptococcus toxins removal by the adsorber, similar to that of myoglobin and bilirubin [79–82]. Beyond static phenotyping, future approaches to HA in septic shock may benefit from integrating concepts of dynamic endotyping, temporal disease trajectories and adaptive treatment strategies, reflecting the evolving nature of sepsis and aligning with emerging precision medicine frameworks.

Clinical course and outcome might be influenced by treatment intensity. Sufficient dosing of HA seems to be reflected by a cumulative volume of blood treated being 13 L/kg body weight or higher [83]. Many studies apparently did not reach this level of treatment intensity. Documentation of patient’s body weight and blood flow rates/treatment times could help to obtain objective treatment dosing information. A frequent concern with unspecific HA is the loss of therapeutic drugs, e.g. antibiotics. Considerable information was gathered regarding drug removal by CytoSorb and certain drug groups were identified that are prone to removal by HA [51, 84]. As far as the particularly important anti-infective agents are concerned, a variety of in-vivo data primarily on the CytoSorb device is available meanwhile, demonstrating a limited effect on the total clearance for many of the tested substances [84]. This applies e.g. for many commonly used ß-lactam antibiotics like meropenem and piperacillin [85, 86], whereas clinically relevant effects have been found for vancomycin, linezolid and azole antimycotics [84, 87, 88]. These important findings should be implemented in the treatment algorithm, e.g. by applying increased loading doses and/or extra doses where deemed necessary [87].

## Conclusions

HA represents a promising adjunctive therapy in the management of septic shock, particularly for patients with hyperinflammatory phenotypes, capillary leak and refractory vasoplegia. However, although the mechanisms - via which HA can be beneficial - are plausible, we are still lacking good quality clinical trials to allow us a definitive judgement on efficacy. Future clinical trials should incorporate careful pheno- and endotype-driven enrichment and pragmatic study designs in bridging the gap between pathophysiological rationale and potential clinical benefit. Furthermore, for clinicians currently using this therapy, participation in registry studies is essential to generate real-world data and bridge the knowledge gap.

## Data Availability

No datasets were generated or analysed during the current study.

## Abbreviations

ADQI: Acute Disease Quality Initiative
ARDS: Acute Respiratory Distress Syndrome
CPFA: Coupled Plasma Filtration Adsorption
CRP: C-reactive protein
CRRT: Continuous Renal Replacement Therapy
DAMPs: Damage-associated molecular patterns
FAS: Fluid Accumulation Syndrome
GIPS: Global Increased Permeability Syndrome
HA: Hemoadsorption
HLA-DR: Human Leukocyte Antigen - DR isotype
IL-6: Interleukin-6
NE: Norepinephrine
PAMPs: Pathogen-associated molecular patterns
TNFR1: TNF receptor-1results
